# Protocol for *in vitro* phase separation of ARID3B proteins

**DOI:** 10.1016/j.xpro.2026.104683

**Published:** 2026-07-02

**Authors:** Wenyu Han, Xiaofeng Li, Junyan Lin, Yongchu Pan

**Affiliations:** 1 Department of Orthodontics, The Affiliated Stomatological Hospital of Nanjing Medical University, Nanjing, China; 2 State Key Laboratory Cultivation Base of Research, Prevention and Treatment for Oral Diseases (Nanjing Medical University), Nanjing, China; 3 Jiangsu Province Engineering Research Center of Stomatological Translational Medicine (Nanjing Medical University), Nanjing, China

**Keywords:** Cell Biology, Genetics, Immunology, Microscopy, Molecular Biology, Protein Biochemistry

## Abstract

Liquid-liquid phase separation (LLPS) of ARID3B is critical in nonsyndromic cleft lip/palate pathogenesis. Here, we present a protocol to visualize ARID3B LLPS. We describe steps for regenerating LLPS with purified protein *in vitro* and detecting ARID3B condensates in mammalian cells. This protocol enables analysis of ARID3B phase separation dynamics and the study of other transcription factors that undergo LLPS.

For complete details on the use and execution of this protocol, please refer to Li et al.^1^

## Highlights

• Generate and purify His6-ARID3B proteins from *E. coli* (Ni-IDA purification)
• Establish a defined *in vitro* LLPS assay for ARID3B condensate formation
• Visualize nuclear ARID3B condensates in HEPM cells using EGFP tagging
• Assess condensate dynamics and protein quality using FRAP and SEC

## Abstract (editor highlights)

Publisher’s note: Undertaking any experimental protocol requires adherence to local institutional guidelines for laboratory safety and ethics.

## Graphical abstract

**Figure:**
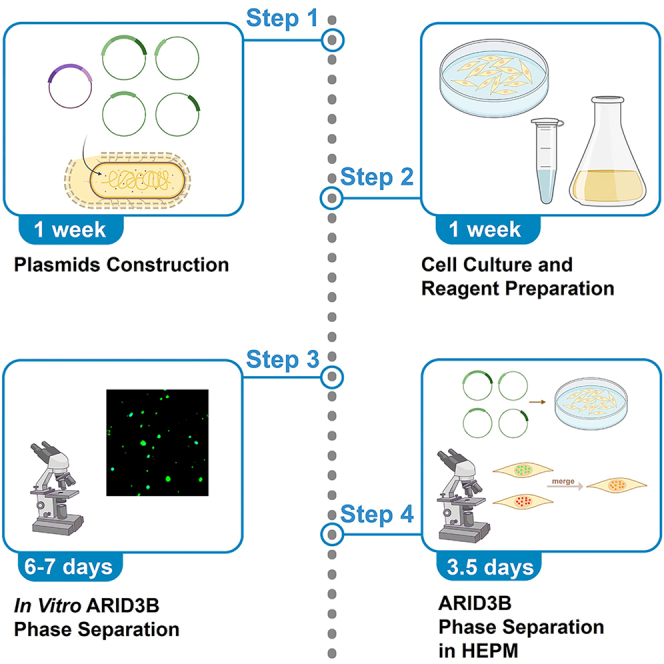

## Before you begin

Liquid-liquid phase separation (LLPS) is a fundamental mechanism governing the spatial organization of biomolecules within cells, facilitating the formation of membraneless condensates that enrich specific proteins and nucleic acids to regulate key biological processes. Many transcription factors undergo LLPS, often mediated by intrinsically disordered regions (IDRs), to compartmentalize transcriptional machinery and regulate gene expression.^2^^,^^3^^,^^4^^,^^5^^,^^6^

ARID3B is a transcription factor implicated in craniofacial development and disease. Bioinformatic analysis predicts the presence of extensive IDRs within ARID3B, suggesting a potential for LLPS. This protocol is designed to biochemically and cellularly characterize the LLPS behavior of ARID3B. It is structured in two sequential tiers: 1) A purified biochemical system to demonstrate phase separation; and 2) In live mammalian cells to observe condensate formation and co-factor recruitment. The protocol is optimized for *E. coli* BL21(DE3) strains and HEPM (Human Embryonic Palatal Mesenchyme) cell line for protein expression.

### Innovation

This protocol integrates biochemical purification, *in vitro* reconstitution, and cell-based imaging to study phase separation of the disease-relevant transcription factor ARID3B.

A key feature of the protocol is the reproducible purification of recombinant ARID3B from the soluble fraction. The protocol provides a practical strategy for expressing and purifying pET28a-His6-ARID3B and pET28a-His6-TEV-EGFP-ARID3B in *E. coli* using low-temperature induction and Ni-IDA affinity chromatography, followed by dialysis into a defined storage buffer suitable for downstream assays.

The protocol also enables systematic mapping of ARID3B condensation domains in cells. By combining full-length and truncation constructs, this workflow identifies the domain requirements for ARID3B condensate formation and for recruitment of SMAD2/3 in the nucleus.

In addition, the protocol provides an integrated analysis of ARID3B phase separation *in vitro* and in cells. It connects biochemical reconstitution, cellular imaging, and FRAP-based assessment of condensate dynamics, allowing direct comparison of ARID3B behavior across experimental settings.

The protocol also incorporates SDS-PAGE, Western blotting, and SEC before LLPS assays to evaluate the purity, identity, and solution-state heterogeneity of purified ARID3B.

Finally, the protocol includes buffer and handling conditions optimized to reduce premature instability. Based on the purification, storage, and SEC results, the protocol uses defined low-temperature handling, PBS-based storage, and controlled reconstitution conditions to reduce precipitation and improve experimental consistency before LLPS assays.

### Institutional permissions

Not applicable for the *in vitro* and standard mammalian cell culture components of this protocol. Researchers must acquire necessary permissions from their local institutional committees for any specific work requiring approval.

### Plasmid construction for mammalian and bacterial expression

**Timing: 1 week**

**Figure 1:**
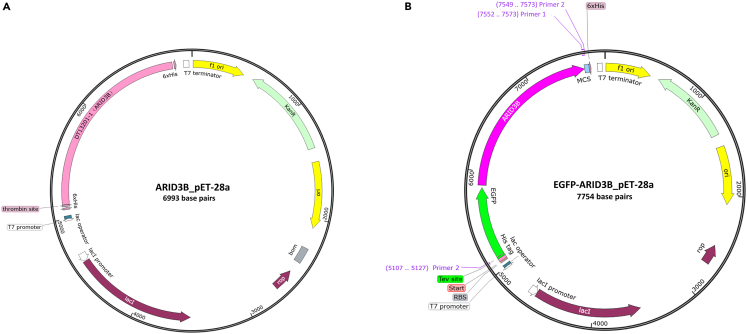
Bacterial expression plasmids for ARID3B Plasmid maps of the bacterial expression constructs used in this study. (A) ARID3B-pET28a, containing full-length ARID3B cloned in frame with an N-terminal 6×His tag. (B) EGFP-ARID3B-pET28a, containing ARID3B fused to EGFP for recombinant expression of the fluorescent fusion protein used in the *in vitro* LLPS assay. Total plasmid sizes are 6,993 bp for ARID3B-pET28a and 7,754 bp for EGFP-ARID3B-pET28a.

**Figure 2:**
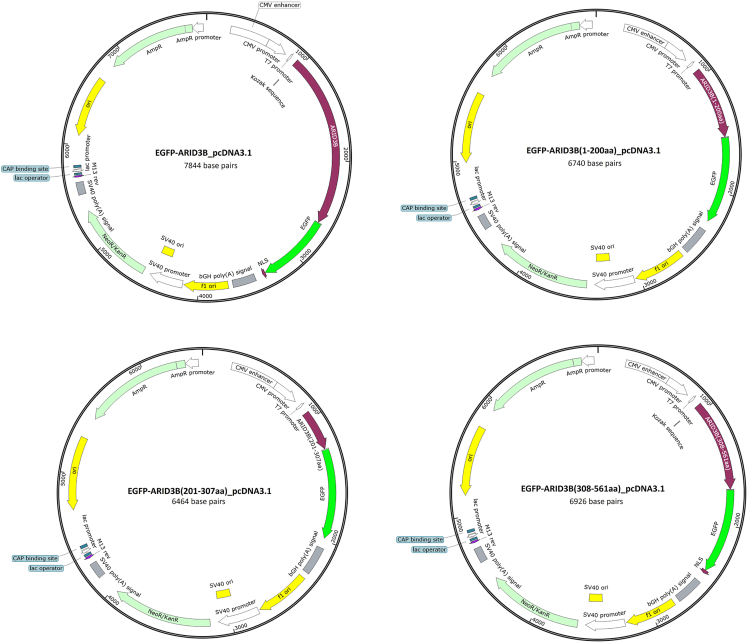
Plasmid map for mammalian expression of ARID3B Schematic representation of the EGFP-ARID3B-pcDNA3.1 construct used for transient expression in mammalian cells. The construct expresses an N-terminal EGFP fusion protein with full-length or truncated ARID3B (IDR1, ARID and IDR2).

This section describes generation of the bacterial expression plasmid for recombinant ARID3B purification and the mammalian expression plasmids for visualization of ARID3B condensates in HEPM cells. Precise molecular cloning and sequence verification are critical for the success of all subsequent steps (Figures 1 and 2).

1. Prepare ARID3B inserts for bacterial and mammalian expression.
  a. Using a validated human ARID3B cDNA as template, amplify the coding sequence (CDS) of full-length ARID3B with a high-fidelity DNA polymerase.
  b. For mammalian truncation constructs, amplify the fragments corresponding to IDR1, ARID, and IDR2. In this study, the truncation boundaries were defined as IDR1 (amino acids 1–200), ARID (amino acids 201–307), and IDR2 (amino acids 308–561).
  c. Design primer pairs that introduce restriction sites compatible with the target vector.
    ***Note:*** For the bacterial construct, use primers containing restriction sites compatible with the multiple cloning site of pET-28a(+), consistent with the plasmid map shown in Figure 1. In our construct, ARID3B was cloned into pET-28a(+) in frame with an N-terminal 6×His tag and a thrombin cleavage site. For the mammalian constructs, use primer pairs compatible with the pEGFP-C1 multiple cloning site (Figure 2).
    **CRITICAL:** Ensure that all inserts are cloned in frame with the upstream tag. Full-length ARID3B must remain in frame with the N-terminal 6×His tag in the bacterial construct and with EGFP in the mammalian constructs.
  d. Perform PCR using annealing temperatures optimized for each primer pair. Verify the expected product sizes by agarose gel electrophoresis and purify the correct bands using a gel extraction kit.
2. Construct the bacterial expression plasmid for recombinant ARID3B purification (Figure 1).
  a. Digest the purified full-length ARID3B PCR product and the pET-28a(+) vector with the appropriate restriction enzymes compatible with the cloning design shown in Figure 1.
  b. Purify the digested insert and vector fragments.
  c. Ligate the ARID3B insert into the linearized vector using T4 DNA ligase. The resulting plasmid is referred to as ARID3B-pET28a.
  d. Transform the ligation mixture into chemically competent *E. coli* cloning cells and plate on LB agar containing kanamycin (50 μg/mL).
  e. Screen positive colonies by colony PCR.
  f. Confirm the correct insertion, orientation, and reading frame by Sanger sequencing across the full ARID3B insert and the His6-ARID3B junction.
    ***Note:*** In the final bacterial expression plasmid, ARID3B is cloned into pET-28a(+) downstream of the T7 promoter/lac operator and in frame with an N-terminal 6×His tag. The plasmid map in Figure 1 also shows a thrombin cleavage site, T7 terminator, kanamycin resistance cassette (KanR), f1 origin, lacI, and the plasmid origin of replication. The total plasmid size is 6993 bp.
  g. Using the same full-length ARID3B PCR product and the same cloning strategy described above, insert ARID3B into the modified pET28a-His6-TEV-EGFP vector to generate EGFP-ARID3B-pET28a.
  h. Screen positive colonies by colony PCR and confirm the correct insertion, orientation, and reading frame by Sanger sequencing across the full ARID3B insert and the His6-TEV-EGFP-ARID3B junction.
  i. Use this construct for bacterial expression and purification of EGFP-ARID3B for the downstream *in vitro* LLPS assay.
3. Construct mammalian expression plasmids for ARID3B and its truncation mutants (Figure 2).
  a. Digest the purified PCR products encoding full-length ARID3B, IDR1, ARID, and IDR2, together with the pEGFP-C1 vector, using the appropriate restriction enzymes.
  b. Purify the digested inserts and vector fragments.
  c. Ligate each ARID3B fragment into the linearized pEGFP-C1 vector using T4 DNA ligase at an approximate 3:1 insert:vector molar ratio overnight at 16°C.
  d. Transform the ligation reactions into chemically competent *E. coli* DH5α cells.
  e. Plate transformed cells on LB agar containing the appropriate selection antibiotic for the mammalian plasmid backbone and incubate overnight at 37°C.
  f. Pick several colonies for each construct and screen them by colony PCR and/or plasmid miniprep followed by restriction digestion.
  g. Purify plasmid DNA from positive clones and verify the inserted sequences and all cloning junctions by Sanger sequencing using vector-specific and internal primers.

***Note:*** In the final mammalian constructs, full-length ARID3B and the truncation mutants are expressed as N-terminal EGFP fusion proteins under the CMV promoter in the pEGFP-C1 backbone for transient transfection and condensate analysis in HEPM cells.

### Cell culture and reagent preparation

**Timing: 1 week**

4. Maintain mammalian cell lines.
  a. Culture HEPM cells in Minimum Essential Medium α supplemented with 10% fetal bovine serum (FBS) and 1% penicillin-streptomycin.
    i. Maintain cells in a humidified incubator at 37°C with 5% CO_2_.
  b. Passage cells every 2–3 days to maintain exponential growth and prevent over-confluence, which can reduce transfection efficiency and cell health.
    i. Aspirate the culture medium from the 100-mm petri dish.
    ii. Wash the cell monolayer gently with 2 to 3 mL of sterile 1× Phosphate-Buffered Saline (PBS) to remove residual serum.
    iii. Add 1 ml 0.25% Trypsin-EDTA solution to cover the cells.
    iv. Incubate the dish at 37°C for 1 to 2 min.
    v. Observe under an inverted microscope. Cells should detach and appear rounded.
      **CRITICAL:** Do not overtrypsinize. Prolonged exposure can damage cells.
    vi. Neutralize the trypsin by adding 4 to 5 mL of complete medium (containing FBS) to the dish.
    vii. Transfer the cell suspension to a sterile centrifuge tube and pellet cells at 100–120 × *g* for 5 min at 20°C–25°C.
    viii. Aspirate the supernatant and resuspend the cell pellet in 1 to 2 mL of fresh, pre-warmed complete medium by gentle pipetting.
    ix. Count cells using a hemocytometer or automated cell counter. Seed new culture flasks or dishes at an appropriate density (3×10^5^–8×10^5^).
5. Prepare all necessary buffers and solutions.
  a. Prepare the buffers for protein purification and *in vitro* LLPS assays according to the exact recipes.
    ***Note:*** The buffers include: Ni-IDA lysis buffer, Ni-IDA equilibration/wash buffer, Ni-IDA elution buffer, PBS (pH 7.4) for dialysis and storage, and the *in vitro* LLPS stock buffer.
    i. Adjust the pH of all buffers at 20°C–25°C using a calibrated pH meter.
    ii. Filter-sterilize buffers that will contact the protein after purification, including PBS and the LLPS stock buffer, using a 0.22 μm filter.
    iii. Store all buffers at 4°C and keep them chilled during protein purification.
      **CRITICAL:** The *in vitro* LLPS buffer should be prepared fresh on the day of the experiment for consistent results.

## Key resources table

**Table:** 

REAGENT or RESOURCE	SOURCE	IDENTIFIER
**Bacterial and virus strains**		

*E. coli* DH5α	Thermo Scientific	Cat# EC0112
*E. coli* BL21(DE3)	Thermo Scientific	Cat# EC0114

**Chemicals, peptides, and recombinant proteins**		

OPTI-MEM	GIBCO	Cat# 31985070
Yeast extract	OXOID	Cat# LP0021
Peptone	OXOID	Cat# LP0137B
Agar powder	Sinopharm	Cat# 10000561
Kanamycin	Sangon Biotech	Cat# A810028-0025
Ampicillin	MedChemExpress	Cat# HY-B0522
Isopropyl β-D-thiogalactopyranoside (IPTG)	Inazco	NA
RIPA buffer	Beyotime	Cat# p0013B
FBS	GIBCO	Cat# 16000044
Trypsin	GIBCO	Cat# 25200072
PBS	Biosharp	Cat# BL302A
SDS	Sinopharm	Cat# 3016642
Tris	Suzhou Yacoo	Cat# S0002
Glycerol	Sigma-Aldrich	Cat# G7893
Imidazole	Sinopharm	Cat# 30104961
BCA Protein Assay Kit	Thermo Scientific	Cat# 23225
NaCl	Sinopharm	Cat# 10019318
Triton X-100	Sigma-Aldrich	Cat# X100
4%paraformaldehyde	Biosharp	Cat# 143174
PEG-4000	Macklin	Cat# 25322-68-3
Lipofectamine 2000	Invitrogen	Cat# 11668019

**Antibodies**		

SMAD2/3 (D7G7) Rabbit Monoclonal Antibody	Cell Signaling Technology	Cat# 8685; RRID:AB_10889933
CoraLite594-conjugated Goat Anti-Rabbit IgG(H+L) (1:100)	Proteintech	Cat No. SA00013-4

**Recombinant DNA**		

pEGFP-C1-ARID3B	Hycyte	NA
pEGFP-C1-IDR1	Hycyte	NA
pEGFP-C1-ARID	Hycyte	NA
pEGFP-C1-IDR2	Hycyte	NA

**Experimental models: Cell lines**		

HEPM	ATCC	CRL-1486TM

**Software and algorithms**		

ImageJ	NIH	https://imagej.nih.gov/
GraphPad Prism 9	GraphPad	https://www.graphpad.com

**Other**		

Dialysis tubing	Viskase Dialysis Tubing	Cat# MD34-14-2

## Materials and equipment

**Table:** Ni-IDA lysis buffer

Reagent	Final concentration	Amount for 100 mL
1 M Tris-HCl, pH 8.0	50 mM	5 mL
5 M NaCl	300 mM	6 mL
1 M imidazole	20mM	2 mL
Triton X-100	1% (v/v)	1 mL
1 M DTT	1 mM	100 μL
100 mM PMSF	N/A	1 mL
ddH_2_O	N/A	To 100 mL

Prepare fresh before use. Keep the buffer at 4°C during protein purification.

**CRITICAL:** PMSF is toxic and should be handled in a chemical fume hood while wearing gloves, a lab coat, and eye protection. DTT and imidazole can cause irritation; avoid inhalation and skin contact. Triton X-100 is harmful if swallowed or inhaled; handle with appropriate personal protective equipment.

**Table:** Ni-IDA equilibration/wash buffer

Reagent	Final concentration	Amount for 100 mL
1 M Tris-HCl, pH 8.0	50 mM	5 mL
5 M NaCl	300 mM	6 mL
1 M imidazole	20mM	2 mL
ddH_2_O	N/A	To 100 mL

Store at 4°C for up to 1 month.

**CRITICAL:** Imidazole can cause irritation. Wear gloves, a lab coat, and eye protection when preparing and handling this buffer.

**Table:** Ni-IDA equilibration/wash buffer

Reagent	Final concentration	Amount for 100 mL
1 M Tris-HCl, pH 8.0	50 mM	5 mL
5 M NaCl	300 mM	6 mL
1 M imidazole	500mM	50 mL
ddH_2_O	N/A	To 100 mL

Store at 4°C for up to 1 month.

**CRITICAL:** Imidazole can cause irritation. Wear gloves, a lab coat, and eye protection when preparing and handling this buffer.

**Table:** In vitro LLPS stock buffer

Reagent	Final concentration	Amount for 100 mL
1 M Tris-HCl, pH 7.4	25 mM	2.5 mL
5 M NaCl	500 mM	10 mL
Glycerol	10% (v/v)	10 mL
1 M DTT	1 mM	100 μL
ddH_2_O	N/A	To 100 mL

Prepare fresh on the day of the experiment or store without DTT at 4°C for up to 1 week. Add DTT immediately before use.

**CRITICAL:** DTT can cause irritation. Wear gloves, a lab coat, and eye protection. This buffer contains high salt and is used for the LLPS assay rather than long-term storage.

## Step-by-step method details

### Expression, purification, and *in vitro* reconstitution of ARID3B phase separation

**Timing:** 6–7 days

This section details the production of recombinant, full-length ARID3B protein from bacteria and the subsequent biochemical demonstration of its intrinsic ability to undergo liquid-liquid phase separation in a defined environment.

1. Expression of Recombinant pET28a-His6-ARID3B in *E. coli* BL21(DE3) (Timing: 2 days).
  a. Transformation and starter culture:
    i. Thaw a 50 μL aliquot of chemically competent *E. coli* BL21(DE3) cells on ice.
    ii. Add 250 ng of the verified ARID3B-pET28a plasmid encoding pET28a-His6-ARID3B to the cells, gently pipette to mix, and incubate on ice for 30 min.
    iii. Heat-shock the cells at 42°C for 90 s in a water bath. Immediately return the tube to ice for 2 min.
    iv. Add 500 μL of pre-warmed, sterile LB medium (without antibiotics) to the tube. Incubate at 37°C for 30 min with shaking at 200 rpm.
    v. Plate 40 μL of the transformation mixture onto an LB agar plate containing kanamycin (50 μg/mL).
    vi. Incubate the plate overnight at 37°C.
    vii. Pick a single colony for immediate downstream culture, and inoculate another single colony into LB medium containing 15%–20% glycerol to prepare a glycerol stock for long-term storage at −80°C.
  b. Small-scale expression test:
    i. Pick a single colony from the transformation plate and inoculate it into 4 mL LB medium containing 50 μg/mL kanamycin.
    ii. Grow the culture at 37°C until OD_600_ = 0.5–0.8.
    iii. Add IPTG to a final concentration of 0.2 mM.
    iv. Incubate the induced cultures under the screening conditions of 15°C and 37°C to compare expression and solubility.
    v. Collect the induced cultures for SDS-PAGE analysis to compare expression level and use the gel result to determine the optimal induction condition for large-scale culture.
  c. Sample preparation for SDS-PAGE analysis:
    i. Centrifuge the induced culture at 13,000 × *g* for 5 min and remove the supernatant.
    ii. Resuspend the pellet in PBS, then add SDS-PAGE loading buffer and heat the sample at 100°C for 10 min.
    iii. Centrifuge briefly and load the supernatant for SDS-PAGE analysis.
    iv. For soluble/insoluble fraction analysis, lyse the whole cells in 20 mM Tris (pH 8.0), 300 mM NaCl, 20 mM imidazole, 1% Triton X-100, 1 mM DTT, and 1 mM PMSF by sonication, then separate the supernatant and pellet for SDS-PAGE detection.
  d. Large-scale culture and induction:
    i. Based on the SDS-PAGE screening result, select the optimal induction condition for large-scale expression.
    ii. Thaw the preserved ARID3B glycerol stock on ice.
    iii. Inoculate 2 μL of glycerol stock into 10 mL LB medium containing 50 μg/mL kanamycin.
    iv. Grow the seed culture overnight at 37°C in a shaking incubator.
    v. Inoculate the seed culture into the large-scale flask at 1% (v/v) with the appropriate antibiotic.
    vi. Grow the culture at 37°C for 3–4 h until OD_600_ = 0.6–0.8.
    vii. For low-temperature induction, pre-cool the culture to 15°C before induction.
    viii. Add IPTG to a final concentration of 0.2 mM.
    ix. Induce expression at the selected condition of 15°C or 37°C, according to the small-scale SDS-PAGE screening result.
    x. Continue induction for 16 h, then harvest the cells by centrifugation and collect the bacterial pellets for purification.
  e. Cell lysis and preparation of the soluble fraction:
    i. Thaw the bacterial pellets on ice and perform all subsequent purification steps at low temperature.
    ii. Resuspend the pellets thoroughly in lysis buffer containing 50 mM Tris (pH 8.0), 300 mM NaCl, 20 mM imidazole, 1% Triton X-100, 1 mM DTT, and 1 mM PMSF.
    iii. Lyse the cells by probe sonication in an ice-water bath for a total of 20 min at 30%–40% power, using 3 s ON/6 s OFF pulses to avoid overheating.
    iv. Clarify the lysate by centrifugation at 13,000 × *g* for 20 min at 4°C and separate the soluble supernatant from the insoluble pellet.
    v. Retain aliquots of the total lysate, supernatant, and pellet for SDS-PAGE analysis.
    vi. Use the clarified soluble supernatant as the input for downstream Ni-IDA affinity purification.
  f. SDS-PAGE analysis of ARID3B expression (Figure 3):
    i. Assemble a 10% SDS-PAGE gel (or 10%–12%, suitable for resolving ARID3B).
    ii. Fill the electrophoresis tank with 1× SDS running buffer.
    iii. Load the following samples into separate wells (typically 10–15 μL per lane): uninduced cells, induced cells (15°C, 16 h), soluble supernatant after lysis, insoluble pellet after lysis and SDS-PAGE Protein Marker in at least one lane.
    iv. Run the gel at 70 V through the stacking gel, then increase to 100 V and continue electrophoresis until the dye front reaches the bottom of the gel (∼60–90 min).
    v. Carefully remove the gel and stain with Coomassie Brilliant Blue for 30–60 min, then destain in destaining buffer until protein bands are clearly visible and background is low.

**Figure 3:**
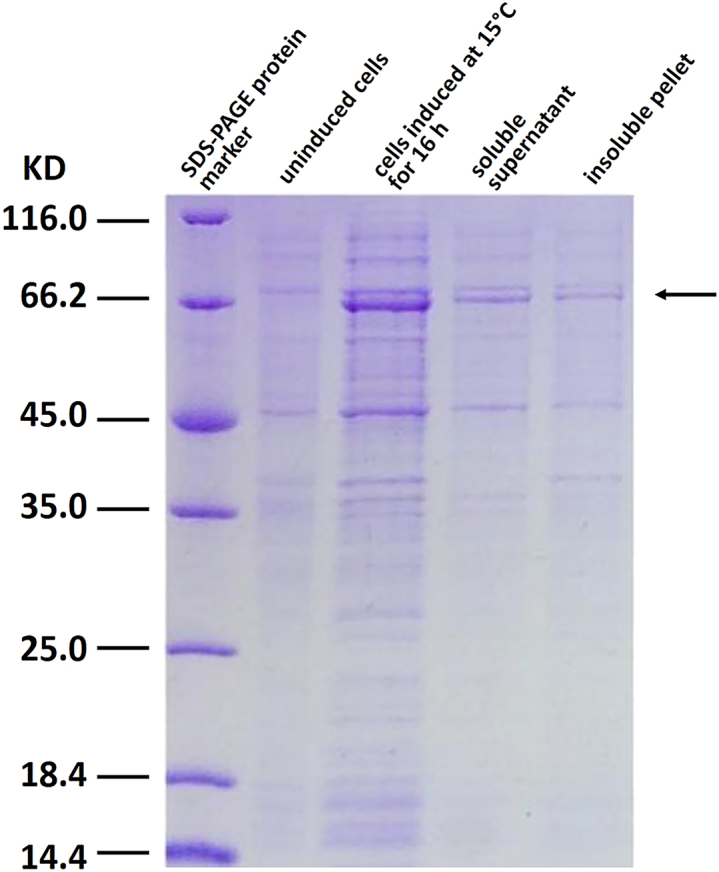
SDS-PAGE analysis of ARID3B expression in *E. coli* BL21(DE3) Coomassie Brilliant Blue-stained SDS-PAGE showing expression and solubility analysis of pET28a-His6-ARID3B in *E. coli* BL21(DE3). Expression was induced in LB medium with 0.2 mM IPTG. Lanes, from left to right: SDS-PAGE protein marker, uninduced cells, cells induced at 15°C for 16 h, soluble supernatant after cell lysis, and insoluble pellet after cell lysis. ARID3B is detected after induction and is present in the soluble supernatant, supporting purification from the soluble fraction.
2. ARID3B purification (Timing: 4 to 5 days)
  a. Ni-IDA affinity purification:
    i. Perform the entire purification procedure at low temperature. Equilibrate the Ni-IDA affinity column with equilibration buffer containing 50 mM Tris (pH 8.0), 300 mM NaCl, and 20 mM imidazole.
    ii. Apply the clarified soluble supernatant to the equilibrated Ni-IDA column and allow binding of pET28a-His6-ARID3B.
    iii. Collect the flow-through after sample loading.
    iv. Wash the column with equilibration buffer to remove non-specifically bound proteins.
    v. Elute the target ARID3B protein using the same buffer supplemented with 500 mM imidazole.
    vi. Collect each elution fraction separately and analyze the fractions by SDS-PAGE.
    vii. Pool the elution fractions with relatively high purity and concentration for downstream processing.
  b. SDS-PAGE analysis of ARID3B purification (Figure 4):
    i. Prepare SDS-PAGE samples from the soluble supernatant after lysis, flow-through after incubation with Ni-IDA resin, and 500 mM imidazole elution fractions, together with a protein marker.
    ii. Perform SDS-PAGE and Coomassie Brilliant Blue staining as described in Step 6.
    iii. Use the SDS-PAGE result to identify the fractions enriched for ARID3B and select the fractions with the highest purity for pooling.

**Figure 4:**
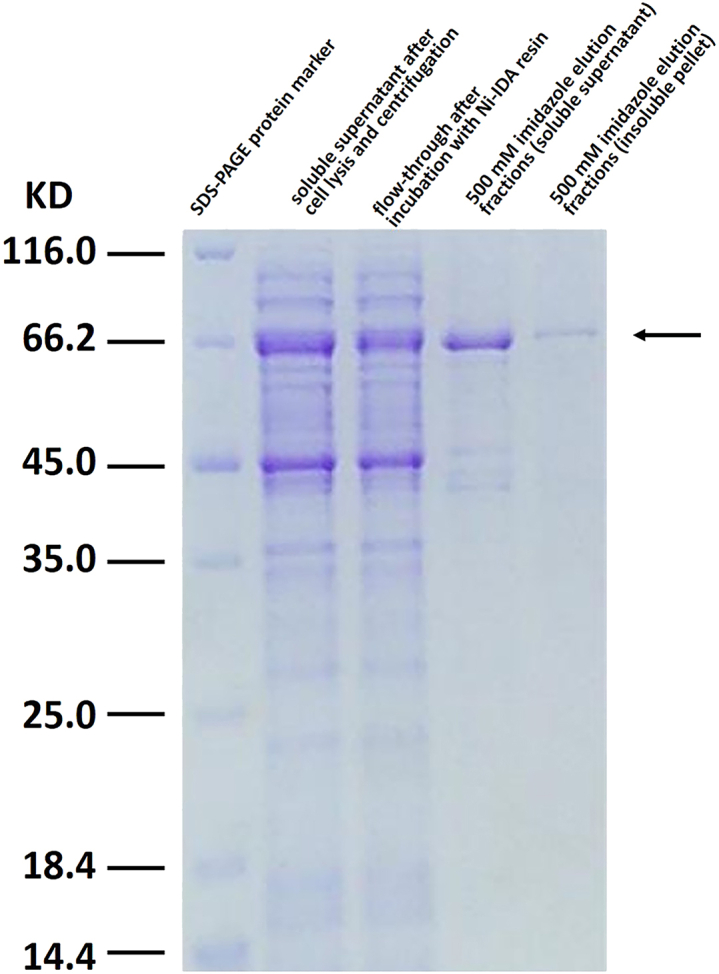
Ni-IDA affinity purification of ARID3B analyzed by SDS-PAGE Coomassie Brilliant Blue-stained SDS-PAGE of fractions collected during Ni-IDA affinity purification of His6-ARID3B from the soluble supernatant. Lanes, from left to right: SDS-PAGE protein marker, soluble supernatant after cell lysis and centrifugation, flow-through after incubation with Ni-IDA resin, 500 mM imidazole elution fractions (soluble supernatant), and 500 mM imidazole elution fractions (insoluble pellet). The ARID3B band is enriched in the 500 mM imidazole elution fractions, consistent with successful purification by Ni-IDA chromatography.
  c. Post-purification processing:
    i. Dialyze the pooled ARID3B fractions into PBS, pH 7.4.
    ii. After dialysis, filter the protein solution through a 0.22 μm filter.
    iii. Aliquot the filtered protein into low-binding tubes for downstream analyses and long-term storage.
  d. Protein concentration determination:
    i. Determine protein concentration using the Bradford assay with BSA as a standard.
    ii. Prepare a series of BSA standards and dilute the ARID3B sample appropriately to fall within the linear range of the assay.
    iii. Measure absorbance according to the Bradford assay protocol and calculate the protein concentration from the BSA standard curve.
  e. Protein quality control:
    i. Analyze the final purified ARID3B sample by SDS-PAGE using BSA (1 μg) as a comparison standard (Figure 5).

**Figure 5:**
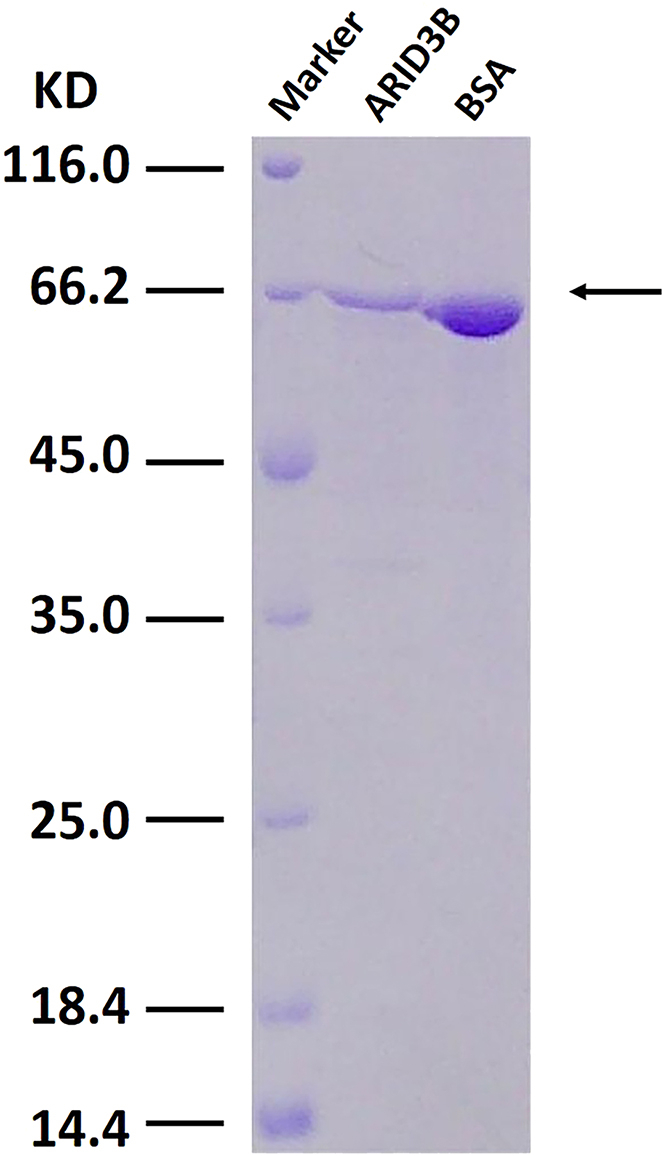
SDS-PAGE quality control of purified ARID3B Coomassie Brilliant Blue-stained SDS-PAGE of the final purified ARID3B preparation. Lanes, from left to right: SDS-PAGE protein marker, purified ARID3B protein, and BSA (1 μg). The purified ARID3B sample showed an apparent purity of approximately 85% by SDS-PAGE.
    ii. Perform Western blot analysis using a multi-tag antibody to confirm the identity of the purified pET28a-His6-ARID3B (Figure 6).

**Figure 6:**
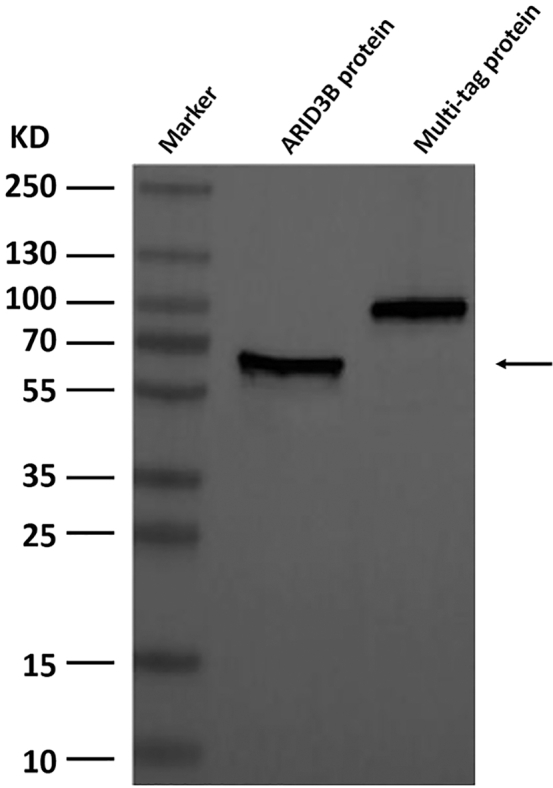
Western blot quality control of purified ARID3B Western blot analysis of the final purified ARID3B preparation. Lanes, from left to right: Western blot marker, purified ARID3B protein, and multi-tag protein control. The positive immunoblot signal in the ARID3B protein lane confirms the identity of purified recombinant pET28a-His6-ARID3B.
  f. Protein storage and stability testing:
    i. Aliquot the purified ARID3B protein into low-binding tubes and store the aliquots at −80°C to avoid repeated freeze-thaw cycles.
    ii. Perform stability testing on representative aliquots to verify the physical stability of the purified protein during storage and handling.
    iii. For the freeze-thaw test, place one aliquot of protein stored at −80°C in an ice-water bath for 5–10 min to allow slow thawing, then keep the thawed protein at 4°C for 0.5 h. Absence of visible abnormalities indicates acceptable freeze-thaw stability.
    iv. For the reconstitution test, slowly add an equal volume of sterile deionized water to the protein at 20°C–25°C, let the sample stand for 5 min, and check for turbidity. If no obvious turbidity or precipitate is observed after brief centrifugation, the reconstitution test is considered acceptable.
    v. Before routine use, thaw one aliquot slowly on ice or in an ice-water bath for 5–10 min, then hold at 4°C for 30 min to confirm that the sample remains clear and free of visible precipitate after thawing.
  g. Size-exclusion chromatography (SEC) analysis of purified ARID3B:
    i. Subject the purified ARID3B protein sample to size-exclusion chromatography (SEC) using an analytical SEC system. Include a blank control and a protein molecular-weight marker mixture to calibrate the retention time range before loading the ARID3B sample.
    ii. Load the purified ARID3B sample onto the SEC column and record the chromatogram under the same running conditions used for the blank and marker samples.
    iii. Identify the major peaks in the ARID3B chromatogram and compare their retention times with those of the marker proteins to estimate the apparent molecular weight of each peak.
    iv. Compare the relative peak areas to evaluate sample heterogeneity.
  h. Expression and purification of EGFP-ARID3B using the same bacterial workflow:
    i. Transform the verified EGFP-ARID3B-pET28a plasmid into the bacterial expression strain and perform recombinant protein expression as described in Major Step One.
    ii. Purify the expressed EGFP-ARID3B protein from the soluble supernatant using the same Ni-IDA affinity purification workflow described above.
    iii. Analyze the purified EGFP-ARID3B fractions by SDS-PAGE and, when needed, by Western blot to confirm enrichment and identity of the fusion protein before use in the downstream *in vitro* LLPS assay.
3. *In vitro* Phase Separation Assay (Timing: 1 day)
  a. Preparation of ARID3B stock solution:
    i. Thaw an aliquot of purified protein on ice.
    ii. Dissolve or dilute the protein in stock buffer.
      ***Note:*** Buffer composed of: 25 mM Tris-HCl (pH 7.4), 500 mM NaCl, 10% (v/v) Glycerol and 1 mM DTT to obtain a concentrated protein stock.
      ***Note:*** For long-term storage, keep the purified protein in a salt-free storage buffer to minimize premature condensation or salt-induced instability.
    iii. Clarify the protein stock by centrifugation at 13,000 × *g* for 10 min at 4°C to remove any aggregates. Transfer the supernatant to a new low-binding tube and keep on ice.
      **CRITICAL:** Always use freshly thawed, clarified protein for phase separation assays to avoid artefacts from aggregates.
  b. Assembly of phase separation reactions:
    i. Prepare a 10% (w/v) PEG-4000 solution in water and filter-sterilize if needed. Keep at 20°C–25°C.
    ii. Add ARID3B into the stock solution, mix with 10% PEG-4000 and water to the corresponding concentration. Gently pipette up and down or flick the tube. Do not vortex.
    iii. Incubate reactions at 20°C–25°C for 15–30 min to allow droplet formation.
      ***Optional:*** Prepare a series of reactions with different ARID3B and PEG-4000 concentrations to determine the phase diagram and threshold for droplet formation.
  c. Microscopy:
    i. Place 3–5 μL of each phase-separation reaction onto a clean glass slide or glass-bottom dish.
    ii. Carefully lower a coverslip onto the sample, avoiding bubbles and excessive pressure.
    iii. For pET28a-His6-TEV-EGFP-ARID3B, image the sample immediately by fluorescence microscopy using 488 nm excitation.
    iv. For pET28a-His6-ARID3B without EGFP, image the sample directly in bright-field mode under the same phase-separation conditions.
    v. Capture representative fields for each condition.

### Visualization of ARID3B phase separation in HEPM cells

**Timing: 3.5 days**

This section details the process of introducing fluorescently tagged ARID3B into HEPM cells, visualizing the formation and dynamics of phase-separated condensates, and validating their functional interaction with the transcriptional coactivator SMAD2/3.

4. Transfection of HEPM Cells (Timing: 1 day)
  a. Cell seeding and coverslip preparation:
    i. Place sterile glass coverslips into the wells of a 6-well culture plate.
    ii. Seed HEPM cells onto coverslips so that they reach 50%–70% confluency on the day of transfection.
    iii. Culture cells overnight in complete growth medium at 37°C with 5% CO_2_.
  b. Transient transfection with Lipofectamine 2000:
    i. For each well, prepare two microtubes:
      Tube A: dilute 2 μg EGFP-ARID3B plasmid DNA in 200 μL OPTI-MEM.
      Tube B: dilute 2 μL Lipofectamine 2000 in 200 μL OPTI-MEM.
    ii. Incubate Tube B for 5 min at 20°C–25°C.
    iii. Combine Tube A and Tube B, mix gently, and incubate for 15–20 min at 20°C–25°C to allow DNA-lipid complex formation.
    iv. Meanwhile, replace the medium on cells with fresh complete medium.
    v. Add the 400 μL DNA-Lipofectamine 2000 mixture dropwise to each well containing cells on coverslips. Gently rock the plate to distribute the complexes evenly.
    vi. Incubate cells for 4 h at 37°C, 5% CO_2_.
    vii. After 4 h, carefully aspirate the transfection medium and replace with fresh complete growth medium.
    viii. Continue incubation for a total of 24 h post-transfection to allow expression and formation of EGFP-ARID3B condensates.
      ***Note:*** Adjust DNA and Lipofectamine 2000 amounts according to well size and cell type if using plates other than 6-well.
5. Live-Cell Confocal Imaging of EGFP-ARID3B Condensates (Timing: 1 day)
  a. Prepare the microscope:
    i. Turn on the confocal laser scanning microscope, the environmental chamber, and the associated software. Allow the system to stabilize for at least 30 min.
    ii. Place a drop of immersion oil on the 100× oil immersion objective.
  b. Image EGFP-ARID3B droplets:
    i. Place the transfected imaging dish on the microscope stage.
    ii. Using the eyepieces or software live view, locate cells expressing a moderate level of EGFP fluorescence. Avoid cells that are overly bright, as this indicates overexpression which can mask phase separation.
    iii. Switch to the confocal acquisition mode. Set the 488 nm laser to a low power (1%–5%) to minimize photobleaching. Set appropriate emission filters for EGFP.
    iv. Focus on the nucleus of a selected cell.
    v. Capture images of multiple cells (n≥10 per condition) from at least three independent transfections.
6. Immunofluorescence for Co-localization with SMAD2/3 (Timing: 1.5 days).
  a. Fix and permeabilize cells: 24–36 hours post-transfection, perform immunofluorescence.
    i. Aspirate the medium and wash cells twice gently with 1× PBS.
    ii. Fix cells by adding 1 mL of 4% paraformaldehyde (PFA) in PBS. Incubate for 15 min at 20°C–25°C.
    iii. Aspirate PFA and wash coverslips 3 times for 5 min each with 1× PBS.
    iv. Permeabilize cells with 0.1–0.3% Triton X-100 in PBS for 10 min at 20°C–25°C.
    v. Wash cells 3 times with PBS to remove Triton.
  b. Blocking and SMAD2/3 immunostaining:
    i. Block non-specific binding by incubating coverslips in normal goat serum for 60 min at 20°C–25°C.
    ii. Dilute the primary antibody against SMAD2/3 in blocking buffer at 1:200.
    iii. Incubate coverslips with anti-SMAD2/3 primary antibody overnight at 4°C in a humidified chamber.
    iv. The next day, wash coverslips 3 times for 5 min with PBS at 20°C–25°C.
    v. Incubate cells with a red-fluorescent secondary antibody diluted in blocking buffer for 1 h at 20°C–25°C in the dark.
    vi. Wash coverslips 3 times with PBS to remove unbound secondary antibody.
  c. Nuclear staining and mounting:
    i. Stain nuclei with DAPI for 5–10 min at 20°C–25°C in the dark.
    ii. Wash coverslips 3 times with PBS.
    iii. Mount coverslips cell-side down onto microscope slides using an anti-fade mounting medium. Seal the edges if necessary.
  d. Imaging and analysis:
    i. Image cells on a fluorescence or confocal microscope using appropriate laser lines and filters.
      ***Note:*** EGFP-ARID3B: 488 nm excitation (green channel). SMAD2/3 (secondary, red): 594 nm excitation (red channel). DAPI: 405 nm excitation (blue channel)
    ii. Acquire single-plane images of transfected cells showing nuclear EGFP-ARID3B.
    iii. Identify and quantify punctate nuclear condensates of EGFP-ARID3B and assess their co-localization with SMAD2/3 in the red channel.

### FRAP of ARID3B condensates *in vitro* and in HEPM cells

7. *In vitro* FRAP of ARID3B condensates (Timing: 1 to 2 h).
  This section measures fluorescence recovery after photobleaching (FRAP) of purified pET28a-His6-TEV-EGFP-ARID3B condensates formed *in vitro* to assess their liquid-like molecular mobility.
  a. Prepare ARID3B condensates for imaging
    i. Assemble *in vitro* phase separation reactions as described in Major Step One, *In vitro* Phase Separation Assay.
    ii. Incubate the reaction for 15–30 min at 20°C–25°C to allow droplet formation.
    iii. Transfer 3–5 μL of the reaction mixture to a glass slide or glass-bottom dish and gently apply a c. coverslip if needed.
    iv. Allow the sample to settle for 2–5 min before imaging.
      ***Note:*** Use a shallow chamber or glass-bottom dish to minimize drift during imaging.
  b. Acquire pre-bleach images
    i. Place the sample on a confocal microscope equipped with a 488 nm laser and FRAP module.
    ii. Use a 100× objective to identify well-separated EGFP-positive droplets.
    iii. Set laser power to the lowest level that provides a clear signal during imaging.
    iv. Acquire 3–5 pre-bleach images at low laser power to establish the initial fluorescence intensity.
    v. Select a region of interest (ROI) within a single droplet. The ROI can be: a small circular region inside the droplet to measure internal exchange, or the entire droplet to measure exchange with the surrounding phase.
    vi. Photobleach the ROI using high-power 488 nm laser illumination for 1–3 s or until fluorescence in the ROI is reduced to approximately 60%–80% of the initial intensity.
      **CRITICAL:** Avoid complete bleaching of the entire field. Excessive bleaching may damage droplets and impair recovery measurements.
  c. Record post-bleach recovery
    i. Immediately after bleaching, record time-lapse images at low laser power.
    ii. Acquire images every 1–3 s for 2–5 min, or until the signal plateaus.
    iii. For slowly recovering droplets, extend the imaging interval and total acquisition time as needed.
8. FRAP of ARID3B condensates in HEPM cells (Timing: 1 day after transfection; 1 to 2 h imaging)
  This section measures FRAP of EGFP-ARID3B nuclear condensates in living HEPM cells to assess molecular exchange in the cellular environment.
  a. Use HEPM cells from Major Step One, perform the steps above to acquire pre-bleach images, photobleach the nuclear condensate and record recovery.

## Expected outcomes

### *In vitro* phase separation (major step one)

This protocol is expected to yield highly purified ARID3B suitable for biochemical LLPS assays. After SDS-PAGE quality control, the final purified ARID3B sample shows a predominant protein band with an apparent purity of approximately 85%. The BSA (1 μg) lane serves as a reference for comparison of protein abundance and band intensity (Figure 5).

In the corresponding Western blot, the ARID3B protein lane produces a clear positive immunoreactive signal, while the multi-tag protein lane serves as the positive control for tag detection. Together, these results confirm that the final preparation contains enriched recombinant pET28a-His6-ARID3B with the expected identity and quality for downstream biochemical assays (Figure 6).

**Figure 7:**
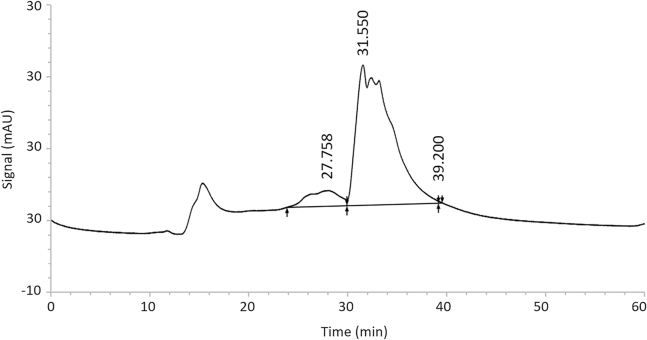
SEC analysis of purified ARID3B SEC chromatogram of purified ARID3B showing a major peak at 31.550 min and a minor peak at 27.758 min.

Analytical SEC of purified ARID3B shows one dominant peak and a smaller minor peak. In the representative sample, the major peak eluted at 31.550 min and the minor peak at 27.758 min, indicating that a single species predominated under the SEC conditions used, with a smaller higher-molecular-weight component also present (Figure 7).

**Figure 8:**
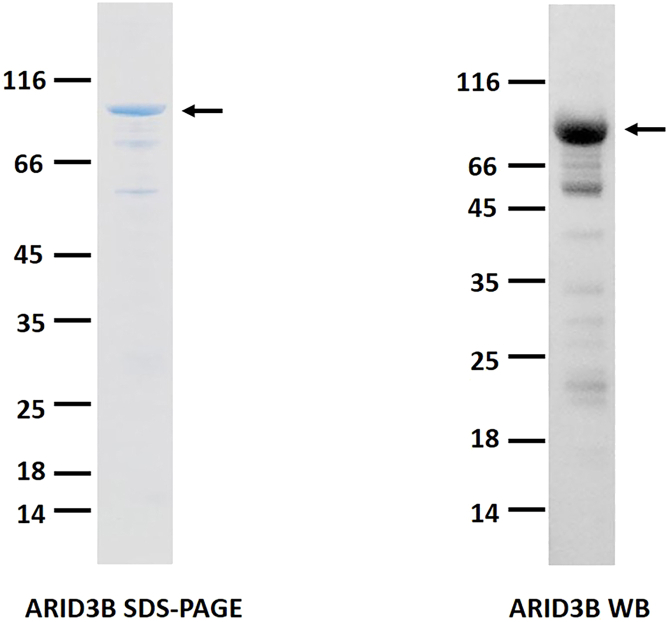
Validation of purified EGFP-ARID3B protein SDS-PAGE and Western blot analysis of purified EGFP-ARID3B showing a major band and corresponding positive signal at ∼75–80 kDa.

Using the bacterial expression and purification workflow described above, the EGFP-ARID3B fusion protein recovers from the soluble supernatant after Ni-IDA affinity purification. On SDS-PAGE, the purified sample shows a prominent at approximately 75–80 kDa, consistent with the predicted size of EGFP-ARID3B. Western blot analysis of the same preparation detects a strong positive immunoreactive band at the same apparent molecular weight, confirming the identity of the purified fusion protein. Together, these results indicate that recombinant pET28a-His6-TEV-EGFP-ARID3B has been enriched to a quality suitable for use in the downstream *in vitro* LLPS assay (Figure 8).

**Figure 9:**
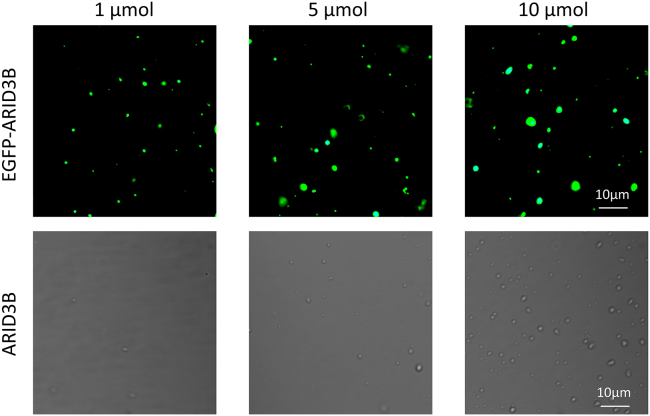
*In vitro* phase separation of purified ARID3B protein Representative images of *in vitro* phase separation of purified ARID3B proteins in LLPS buffer containing PEG-4000. EGFP-ARID3B forms round condensates visible in the fluorescence channel. Purified ARID3B without EGFP also forms spherical condensates visible by bright-field imaging under the same assay conditions. Scale bars: 10 μm.

When the purified EGFP-ARID3B is diluted into the LLPS stock buffer (25 mM Tris-HCl pH 7.4, 500 mM NaCl, 10% glycerol, 1 mM DTT) and mixed with PEG-4000 as a crowding agent, samples that exceed the phase-separation threshold (>1 μmol) become visibly more turbid compared with protein-only controls. For EGFP-ARID3B, fluorescence microscopy reveals numerous round, EGFP-positive droplets with smooth boundaries. For non-EGFP ARID3B, bright-field imaging alone reveals similar spherical condensates under the same assay conditions. Together, these observations indicate that droplet formation is observed both with the EGFP-tagged construct and with the non-EGFP ARID3B preparation, supporting that the condensates are not solely an artifact of fluorescent tagging. Droplet number and size vary with EGFP-ARID3B concentration and PEG-4000 percentage, consistent with the concentration-dependent LLPS behavior of ARID3B (Figure 9).

### FRAP analysis of ARID3B condensates in HEPM cells and *in vitro*

**Figure 10:**
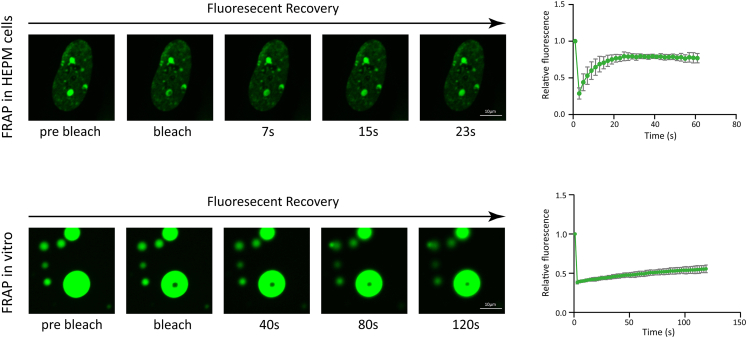
FRAP analysis of ARID3B condensates in HEPM cells and *in vitro* Representative FRAP images and recovery curves of EGFP-ARID3B condensates in HEPM cells (top) and *in vitro* (bottom). A defined region within each condensate was photobleached, and fluorescence recovery was monitored over time. Scale bars: 10 μm.

In both live-cell and *in vitro* FRAP experiments, EGFP-ARID3B condensates show partial fluorescence recovery after photobleaching, consistent with dynamic molecular exchange within the condensates. Liquid-like ARID3B condensates show partial or substantial fluorescence recovery within tens of seconds to a few min, indicating internal molecular mobility (Figure 10).

### Cellular phase separation in HEPM cells (major step two)

In HEPM cells transfected with EGFP-ARID3B full-length (FL), ARID3B localizes predominantly to the nucleus and forms discrete, punctate condensates rather than a homogeneous nuclear signal. These nuclear condensates are stable and can be readily distinguished from diffuse background.

For the EGFP-ARID3B truncation constructs, the expected patterns are:

IDR2-containing constructs: robust nuclear puncta consistent with condensate formation.

IDR1 or ARID domain alone: predominantly diffuse nuclear or nucleo-cytoplasmic distribution with markedly reduced or absent puncta.

**Figure 11:**
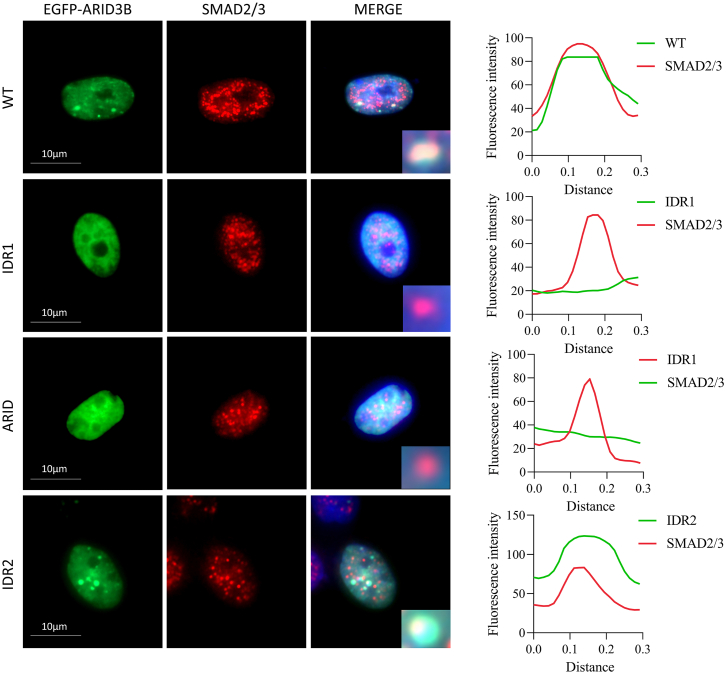
Cellular phase separation of ARID3B and co-localization with SMAD2/3 Confocal microscopy images of HEPM cells transfected with EGFP-ARID3B full-length and the indicated domain truncation mutants (IDR1, ARID, IDR2). Columns show the EGFP signal (green), immunofluorescence signal for endogenous SMAD2/3 (red), and the merged image highlights co-localization. Scale bars: 10 μm.

Following immunostaining, endogenous SMAD2/3 partially co-localizes with EGFP-ARID3B condensates, producing overlapping green (EGFP) and red (SMAD2/3) signals in merged images and supporting an LLPS-mediated transcriptional regulation mechanism in craniofacial cells. DAPI staining outlines the nuclear compartment. Together, these outcomes indicate that ARID3B undergoes LLPS both *in vitro* and in HEPM cells, and that the IDR2 domain is sufficient to drive condensate formation and recruit SMAD2/3 (Figure 11).

## Limitations

The defined LLPS buffer (25 mM Tris-HCl pH 7.4, 500 mM NaCl, 10% glycerol, 1 mM DTT, PEG-4000) recapitulates only a subset of the factors influencing ARID3B behavior in the nucleus. It lacks nucleic acids, partner proteins, crowding from the full nuclear proteome, and post-translational modifications. Consequently, phase boundaries and droplet properties measured *in vitro* may differ quantitatively from those in vivo, and condensate responses to stimuli in cells may not be fully captured in this reconstituted system.

Full-length ARID3B shows limited solubility during bacterial expression and therefore requires low-temperature induction and careful handling throughout purification. Following Ni-IDA affinity purification and buffer exchange into PBS (pH 7.4), the protein can be obtained at a quality suitable for downstream LLPS assays. However, SDS-PAGE, Western blot, and SEC analyses indicate that the purified preparation is not fully homogeneous in solution. Therefore, although the protocol yields usable recombinant ARID3B for phase-separation experiments, a minor heterogeneous population may still be present.

HEPM cells provide a tractable and disease-relevant model for craniofacial mesenchyme, but they do not fully recapitulate primary human cranial neural crest cells or in vivo craniofacial tissues. Differences in chromatin landscape, signaling context, and expression of co-factors may influence the exact composition, dynamics, and functional outcomes of ARID3B condensates. Extrapolation of LLPS behavior observed in HEPM cells to the full spectrum of nsCL/P-relevant cell types should therefore be made with caution.

This protocol includes fluorescence imaging, bright-field imaging, and FRAP analysis to assess ARID3B condensates *in vitro* and in cells. These assays support droplet formation and dynamic exchange, but they do not provide a complete description of condensate material properties such as viscosity, internal organization, or molecular stoichiometry. More detailed biophysical analyses would be needed to fully define the physical state of ARID3B condensates.

## Troubleshooting

### Problem 1

Low solubility and yield of ARID3B during bacterial expression and purification (related to Step 1b, 1d and 1f).

### Potential solution

• During expression screening, compare 15°C and 37°C induction and use the condition that gives the best soluble fraction by SDS-PAGE.
• For large-scale culture, maintain induction at 15°C for 16 h if this condition improves solubility.
• Keep all samples and buffers cold during lysis and purification, and use short sonication pulses with adequate cooling intervals.
• Prepare lysis buffer fresh and include 1 mM PMSF and 1 mM DTT to reduce degradation and oxidation.
• If solubility remains poor, reduce expression level by lowering IPTG concentration or shortening induction time during the screening step.

### Problem 2

Poor binding of ARID3B to the Ni-IDA column or substantial loss of protein in the flow-through (related to Step 2a and 2b).

### Potential solution

• Confirm that the Ni-IDA column is equilibrated with 50 mM Tris (pH 8.0), 300 mM NaCl, and 20 mM imidazole before loading.
• Make sure the clarified supernatant is free of particulates before loading.
• Retain and analyze the flow-through by SDS-PAGE to assess whether a large fraction of ARID3B failed to bind.
• If binding is poor, reduce the sample load or perform a second round of binding with fresh resin.
• Check that the plasmid sequence and N-terminal His-tag junction were correctly verified during cloning.

### Problem 3

No phase separation observed *in vitro* even with high protein concentration (related to Step 3a, 3b and 3c).

### Potential solution

• Prepare fresh 10% (w/v) PEG-4000 solution, confirm correct concentration by weight, and avoid repeated freeze-thaw cycles.
• Systematically vary ARID3B concentration and PEG-4000 percentage in the LLPS assay.
• Confirm that the protein remains clear after thawing and before use.
• Check the sample by SDS-PAGE and SEC before setting up the LLPS assay.
• Use both fluorescence and bright-field imaging to confirm whether spherical droplets form under the tested conditions.

### Problem 4

EGFP-ARID3B appears diffusely nuclear without forming visible condensates in cells (related to Step 4b, 5a and 5b).

### Potential solution

• Reduce plasmid DNA input while keeping Lipofectamine 2000 constant during transfection.
• Image cells 24 h post-transfection, when expression is sufficient but not extreme, rather than very early or very late time points.
• Ensure HEPM cells are healthy and 50%–70% confluent at transfection; avoid over-confluent or serum-deprived conditions.
• Verify that the correct construct (full-length or IDR2-containing) is used in conditions where condensates are expected, as IDR1-or ARID-only constructs may remain largely diffuse.

### Problem 5

Weak or absent SMAD2/3 signal in immunofluorescence (related to Step 6a–6d).

### Potential solution

• Confirm that 4% PFA fixation for 10–15 min and 0.1%–0.3% Triton X-100 permeabilization for 10 min provide adequate staining.
• Optimize primary antibody dilution and incubation time, for example overnight at 4°C versus 2 to 3 h at 20°C–25°C.
• Include BSA or serum in the blocking and antibody diluent to reduce background and improve signal.
• Check that secondary antibody species and fluorophore are correctly matched to the primary antibody and imaging channels.

### Problem 6

Weak, unstable, or no fluorescence recovery after photobleaching in FRAP (related to Optional Step).

### Potential solution

• Confirm that the pre-bleach signal is sufficiently strong and that only a defined region of interest is bleached, avoiding excessive bleaching of the entire droplet or surrounding area.
• Reduce bleaching duration or laser power if the fluorescence signal is completely lost or if the structure appears damaged after bleaching.
• Select round, well-separated droplets or condensates with minimal movement during imaging, and allow the sample to stabilize before starting the FRAP experiment.
• Include an unbleached reference region and background region for normalization to correct for acquisition-related bleaching and signal drift.
• If no recovery is observed, repeat the experiment with freshly prepared protein samples or cells with moderate expression levels, as irreversible aggregation or overexpression can reduce molecular mobility.

## Resource availability

### Lead contact

Further information and requests for resources and reagents should be directed to and will be fulfilled by the lead contact, Yongchu Pan (panyongchu@njmu.edu.cn).

### Technical contact

Technical questions on executing this protocol should be directed to and will be answered by the technical contact, Xiaofeng Li (xfli@stu.njmu.edu.cn).

### Materials availability

Plasmids generated in this study, including the EGFP-ARID3B-pcDNA3.1 series, ARID3B-pET28a, and EGFP-ARID3B-pET28a, are available from the lead contact upon completion of a standard material transfer agreement (MTA). This study did not generate new unique biological materials.

### Data and code availability

The raw imaging data and representative datasets supporting the expected outcomes in this protocol are available from the lead contact upon reasonable request. This protocol does not involve the use of original code.

## Acknowledgments

We thank all members of the Pan laboratory for their constructive discussions. This work was supported by the Jiangsu Province Capability Improvement Project through Science, Technology and Education-Jiangsu Provincial Research Hospital Cultivation Unit (YJXYYJSDW4) and Jiangsu Provincial Medical Innovation Center (CXZX202227).

## Author contributions

Conceptualization, X.L. and J.L.; methodology, X.L. and W.H.; sampling and data curation, W.H., X.L., and J.L.; investigation, W.H. and X.L.; writing – original draft preparation, W.H.; writing – review and editing, Y.P. and X.L.; funding acquisition, Y.P.; supervision, Y.P.

## Declaration of interests

The authors declare no competing interests.
